# Highly luminescent organic-inorganic hybrid antimony halide scintillators for real-time dynamic and 3D X-ray imaging

**DOI:** 10.1038/s41377-025-02152-x

**Published:** 2026-01-26

**Authors:** Haixia Cui, Wanjiao Li, Qianxi Li, Shaolong Wang, Mingye Zhu, Yongjing Deng, Shujuan Liu, Qiang Zhao

**Affiliations:** 1 State Key Laboratory of Flexible Electronics (LoFE) & Institute of Advanced Materials (IAM), Nanjing University of Posts & Telecommunications, 9 Wenyuan Road, Nanjing, 210023 Jiangsu China; 2 School of Physics and Optoelectronic Engineering & School of Chemistry and Materials Science, Nanjing University of Information Science and Technology, Nanjing, 210044 Jiangsu China

**Keywords:** Metamaterials, Imaging and sensing

## Abstract

Real-time dynamic and three-dimensional (3D) X-ray imaging are the most challenging types of X-ray imaging technology, placing more rigorous standards on scintillators. Lead-based (Pb^2+^) organic-inorganic hybrid halide (OIHH) scintillators with high X-ray absorption coefficients have been demonstrated to exhibit excellent scintillation performance. However, their toxicity and instability hindered further development, and it is necessary to explore novel low-toxic metal-based OIHHs possessing excellent scintillation performance. Antimony-based (Sb^3+^) OIHHs are not only environmentally friendly, but also show good stability compared to Pb^2+^-based OIHHs, which make them promising candidates as excellent scintillators. Currently, the understanding of Sb^3+^-based OIHH scintillators for X-ray detection and imaging is still in infancy and requires further exploration. Herein, we designed two Sb^3+^-based OIHH crystals of (BPP)_2_SbCl_5_ (CP1) and (BPP)_2_SbCl_5_ 0.5 H_2_O (CP2), which have very similar crystal structures except the introduction of water molecules in CP2. Experimental and theoretical results reveal that CP2 has larger lattice distortion and smaller freedom of motion, which can promote the self-trapped excitons emissions. A flexible scintillator screen based on CP2 crystals was prepared and applied for real-time dynamic and 3D X-ray imaging, which is the first time for Sb^3+^-based OIHH scintillators and significantly broadens the potential of Sb^3+^-based OIHH scintillators.

## Abstract (web summary)

We enhanced the scintillation performance of Sb³⁺-based halides by introducing H_2_O to enhance lattice distortion and restrict free degrees, and realized real-time and 3D X-ray imaging using the flexible film.

## Graphical abstract

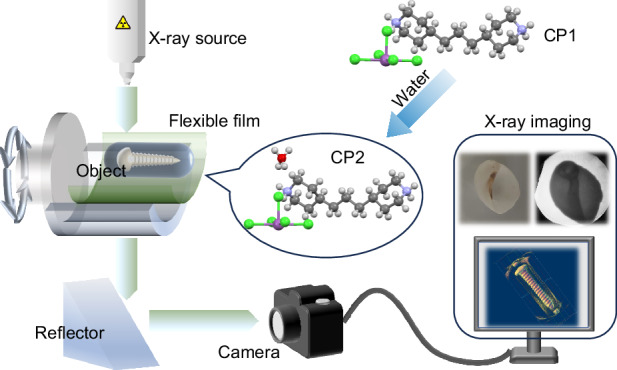

## Introduction

In recent years, X-ray detection and imaging technology has been widely applied in many important fields, such as medical diagnosis, device inspection, material sciences, and garnered increasing attention from researchers^1–3^. Real-time dynamic and three-dimensional (3D) X-ray imaging, which are crucial for examining the intricate internal structures of complex materials, are the most challenging types of X-ray imaging technology^4–8^. Scintillator screens based on rigid flat panels applying to these advanced X-ray imaging techniques usually suffer from image distortion and vignetting problems in imaging of nonplanar and irregular objects due to the uneven spatial distribution of X-ray dose^9–11^. These problems can be effectively solved with the help of flexible scintillator screens, which could seamlessly adhere to the irregular objects^12^. Therefore, there is an urgent need to develop flexible scintillator screens based on high-performance X-ray scintillators for real-time dynamic and 3D X-ray imaging.

It is well known that lead-based (Pb^2+^) organic-inorganic hybrid halide (OIHH) scintillators with highly stereo active lone pairs (ns^2^ electron configuration) have been a hot research topic because of their high X-ray absorption coefficients and great processibility^13–16^. However, their toxicity and instability impede their further development. Therefore, it is urgent to look for other novel low-toxic alternatives that possess ns^2^ electron configuration, such as Sn^2+^, Ge^2+^ and Sb^3+^-based OIHHs, which exhibit large Stokes shifts and broad emissions due to the strong electron-phonon coupling^17–19^. Among them, Sb^3+^-based OIHHs are environmentally friendly compared to Pb^2+^-based OIHHs, but also show large atomic numbers compared to Ge^2+^ and Sn^2+^-based OIHHs^20–22^. In addition, Sb^3+^-based OIHHs also have the advantages of simple preparation and high stability. Therefore, Sb^3+^-based OIHHs are the promising candidates for next generation scintillators. However, the understanding of Sb^3+^-based OIHH scintillators for X-ray detection and imaging is still in early stage and requires further exploration of high-performance Sb^3+^-based scintillators.

Herein, we designed and synthesized two 0D Sb^3+^-based OIHH crystals (BPP)_2_SbCl_5_ (CP1) and (BPP)_2_SbCl_5_ 0.5 H_2_O (CP2) (BPP = 1,3-bis(4-piperidyl) propane) as scintillators. They have very similar crystal structures except the introduction of water molecules in CP2. Experimental and theoretical results reveal that CP2 crystals have larger lattice distortion and smaller freedom of motion than CP1, which can promote the self-trapped excitons (STE) emissions and inhibit the nonradiative decay. Thus, CP2 crystals show a higher photoluminescence quantum yield (PLQY) of 97.25% than that of CP1 (73.38%). Benefitting from this, CP2 crystals show a high light yield of 32332 photons MeV^–1^. In addition, we have successfully developed a flexible scintillator screen utilizing CP2 crystals, and investigated its potential in real-time dynamic and 3D X-ray imaging. To our knowledge, this work is the first time for Sb^3+^-based OIHHs to realize real-time dynamic and 3D X-ray imaging, which makes a great contribution to the development of Sb^3+^-based OIHHs.

## Results

### Structure characterizations

High PLQYs of Sb^3+^-based OIHHs is a necessary prerequisite for their high light yields^23^. Previously, we found that their PLQYs closely correlates with their Sb•••Sb distance. Specifically, a larger value of the Sb•••Sb distance ( > 8 Å) tends to result in a higher PLQY^24–26^. For clarity, the Sb•••Sb distance mentioned herein specifically refers to the shortest distance between adjacent antimony atoms. However, some other factors (structural stability, heavy atom effect, coordination environment, etc.) also affect the final result of PLQYs^27,28^. If Sb^3+^-based OIHHs possessing identical Sb•••Sb distances, the influence of other factors on their PLQYs can be explored.

**Fig. 1:**
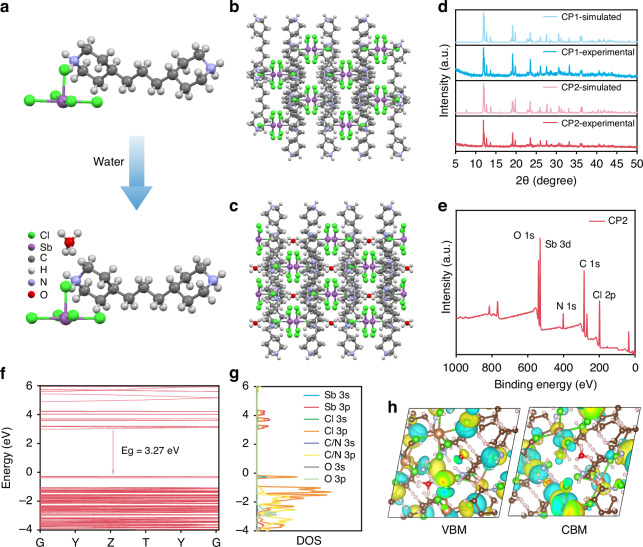
Structural and electronic characterizations of Sb^3+^-based OIHHs. **a** Schematic diagram of the introduction of water molecules in CP2 crystals; **b** Crystal structures of CP1 and **c** CP2; **d** PXRD of CP1 and CP2; **e** XPS of CP2; **f** Electronic band structure and **g** DOS of CP2; **h** VBM (left) and CBM (right) of CP2

Thus, CP1 and CP2 crystals (CCDC: 2098042 and 2404330) were obtained via a typical method of cooling crystallization, and the detailed synthesis procedures were described in Methods part. The crystal structures of CP1 and CP2 were determined through the test of single-crystal X-ray diffraction (SCXRD), and the comprehensive crystallographic data were presented in Tables S1–S5. CP1 and CP2 crystals exhibit similar 0D geometry at the molecular level, and their Sb•••Sb distances are almost the same, which are 8.210 and 8.212 Å, respectively. The largest structural difference between them is the introduction of H_2_O in CP2 (Fig. 1a), thus causing some differences in crystal structures. For example, the symmetry of crystals has been decreased, as shown in Fig. 1b, c, CP1 crystallizes at *Fddd* space group, while CP2 crystallizes at *Fdd2* space group. Besides that, a large lattice distortion of CP2 crystals is formed, which can be quantified by the bond angle variance (*σ*^*2*^) and bond length distortion (Δ*d*) (Eqs. 1 and 2^)^^29^.

1 $${\sigma }^{2}=\frac{1}{7}{\sum }_{{\rm{i}}=1}^{8}{({\alpha }_{i}-{90}^{0})}^{2}$$

2 $$\Delta d=\frac{1}{5}{\sum }_{n=1}^{5}{\left(\frac{{d}_{n}-d}{d}\right)}^{2}$$

Here, *α*_*i*_ denotes the Cl-Sb-Cl bond angle, while *d*_*n*_ and *d* represent the individual and average Sb-Cl bond length, respectively. From the above equations, we can know that CP2 crystals show larger bond angle variance and bond length distortion (*σ*^*2*^ = 12.268 and Δ*d* = 1.2842 × 10^–3^) than CP1 crystals (*σ*^*2*^ = 11.317 and Δ*d* = 1.21 × 10^–3^). The reduced symmetry and increased lattice distortion can enhance the electron-phonon coupling and are beneficial to the formation of exciton self-trapping^30^.

To validate the phase purity of CP1 and CP2 single crystals, the powder X-ray diffraction (PXRD) measurements were performed. As shown in Fig. 1d, the diffractions of experimental values match well with the simulated results, showing high phase purity. To verify the chemical states of the elements in CP1 and CP2 single crystals, the related X-ray photoelectron spectroscopy (XPS) measurements were further performed (Fig. 1e, Fig. S1 and Fig. S2). Figure 1e shows the typical characteristic peaks of Sb 3 d, and the binding energy of 540.2 and 530.9 eV correspond to Sb 3d_3/2_ and 3d_5/2_, respectively. The characteristic O 1 s peaks are located at 67.9 eV, illustrating the coexistence of O atoms. To validate the thermal stability of CP1 and CP2, the thermogravimetry analyses (TGA) are conducted and the curves in Figure. S3 illustrate that they have good thermal stability.

To achieve further insight into the structure-property relationships of CP1 and CP2, density functional theory (DFT) calculations were conducted. As shown in Fig. S4a and Fig. 1f, CP1 and CP2 crystals both exhibit flat band dispersion for valence and conduction bands. This characteristic suggests negligible coupling effects between adjacent [SbCl_5_]^2-^ square-pyramids, indicating that the excitons are confined in [SbCl_5_]^2-^ clusters. The band gaps of CP1 and CP2 are 3.36 eV and 3.27 eV, respectively, which are regarded as indirect band gaps and are consistent with experimental values. In addition, the partial density of states (DOS) of CP1 and CP2 crystals are displayed in Fig. S4b and Fig. 1g. For CP1 crystals, we can observe that the p orbitals of Cl and Sb atoms significantly contribute to the conduction band minimum (CBM), while the p orbitals of Cl atoms and organic molecules contribute to the valence band maximum (VBM). The orbital contribution of CP2 crystals is similar to that of CP1, except that the p orbitals of O atoms also have contribution to VBM. Besides that, Fig. 1h and Fig. S4 show charge density distributions of VBM and CBM of CP1 and CP2 crystals, which are mainly localized in [SbCl_5_]^2-^ clusters. Thus, the photoluminescence (PL) emissions of CP1 and CP2 crystals are mainly originated from electron transitions occurring in [SbCl_5_]^2-^ clusters^31,32^.

### Photophysical properties

**Fig. 2:**
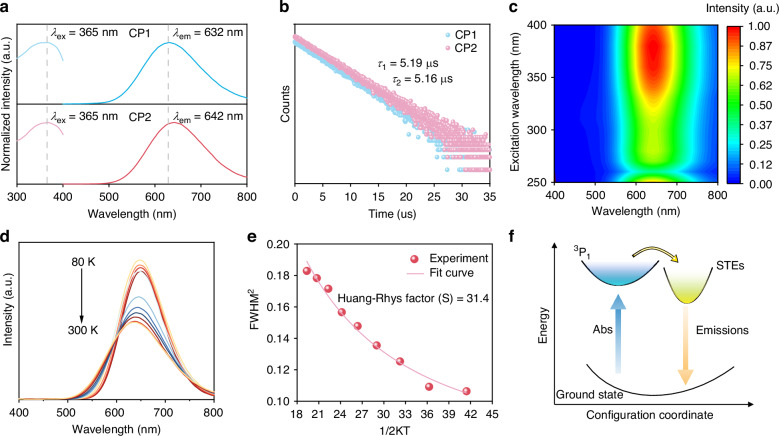
PL characterizations of Sb^3+^-based OIHHs. **a** PL and PLE curves of CP1 and CP2 crystals; **b** PL decay curves of CP1 and CP2 crystals; **c** Excitation-wavelength dependent PL mapping of CP2 crystals; **d** Temperature-dependent PL spectra of CP2 crystals; **e** The functional plot between FWHM and temperature; **f** The PL mechanism of CP2 crystals

In order to elucidate the photophysical properties of CP1 and CP2 crystals, systematic and comprehensive optical tests were conducted. Figure S5 displays the UV-Vis absorption spectra of CP1 and CP2 crystals, and the main absorption regions of them are from 200 to 400 nm. Using the Tauc-plot method, we obtained the band gaps of 3.26 eV and 3.13 eV of CP1 and CP2, respectively. The experiment data are consistent with the above-simulated results. The photoluminescence excitation (PLE) and PL spectra of CP1 and CP2 crystals are shown in Fig. 2a. Under excitation at 365 nm, CP1 crystals exhibit a broadband orange emission at 632 nm, a full width at half-maximum (FWHM) of 144 nm and a Stokes shift of 267 nm, while CP2 crystals exhibit an orange emission at 642 nm, a wide FWHM of 146 nm and a large Stokes shift of 277 nm. Both of them show large Stokes shifts, which can alleviate self-absorption and is beneficial for the light output. Figure 2b shows the PL decay curves for CP1 and CP2 crystals at room temperature, and the decay lifetimes (*τ*) are measured to be 5.19 and 5.16 µs, respectively. The PLQYs of CP1 and CP2 were measured to be 73.38% and 97.25% in Fig. S6, respectively. The detailed photophysical parameters of CP1 and CP2 crystals have been listed in Table S6. The radiative transition rates (*k*_*r*_) and nonradiative transition rates (*k*_*nr*_) were calculated with the following equations (Eqs.(3) and (4))^33^. CP2 crystals have a weaker nonradiative transition rate (0.0053) than CP1 crystals (0.0513), which can be attributed to the introduction of water molecules to inhibit non-radiative decay. The above photophysical descriptions of CP2 crystals, such as efficient broadband emissions, large Stokes shifts, and microsecond-level lifetimes, imply that their emissions might belong to the self-trapped excitons (STEs)^34^.

3 $${k}_{r}=\frac{{PLQY}}{\tau }$$

4 $${k}_{{nr}}=\frac{1}{\tau }-{k}_{r}$$

To further investigate the luminescence behaviors of CP2 crystals, excitation-dependent and temperature-dependent PL tests have been conducted. Figure 2c reveals the emissions under different PLE excitation wavelengths of CP2 crystals, and the peaks centered at 642 nm have a negligible split and shift. This phenomenon indicates that the radiative recombination of CP2 crystals originates from a single radiative pathway. Figure 2d displays the temperature-dependent PL spectra of CP2 crystals and the PL emissions blue shift from 642 nm to 632 nm during the heating process. In addition, the PL intensity firstly increases slightly (80-140 K) and then continuously decreases (Fig. S7), indicating that there is an energy barrier (Δ*E*) between the excited state and STEs state. The energy barrier can be overcome with the help of thermal activation, which can be evaluated from the following Eq.(5) and the final result of *ΔE* is 12.06 meV^35^.

5 $$\varDelta E={k}_{B}T$$

where *k*_*B*_ is the Boltzmann constant. During the heating process, the FWHM of CP2 crystals gradually broadened due to the progressively enhanced electron-phonon coupling, which can be quantified by Eq. 6^36^.

6 $${FWHM}=2.36\sqrt{S}{\hbar \omega }_{{phonon}}\sqrt{\coth \frac{{\hbar \omega }_{{phonon}}}{2{k}_{B}T}}$$

where *ħω*_*phonon*_ represents phonon frequency. Generally, the value of *S* is positively correlated with electron-phonon interaction. *S* in Eq.(6) is fitted as 31.4 (Fig. 2e), which is higher than most of the reported Sb^3+^-based OIHHs, such as TEBA-2 (S = 26.69), MTP_2_SbCl_5_ (S = 22.20), etc., indicating strong electron-phonon coupling for CP2 crystals^10,27^. Besides that, the contributions of electron-phonon coupling have been quantified by Eq. 7^37^.

7 $$\varGamma \left(T\right)={\varGamma }_{0}+\frac{{\varGamma }_{{L}_{0}}}{\exp \left(h{\omega }_{{phonon}}/{k}_{B}T\right)-1}$$

where *Γ*_*0*_ is the FWHM at 0 K, and *Γ*_*L0*_ represents the contributions of electron-phonon coupling. *Γ*_*L0*_ is fitted as 88 meV (Fig. S8), larger than that of CP1 (65 meV), [DMPZ]_2_SbCl_6_·Cl·(H_2_O)_2_ (65.65 meV), etc., verifying strong electron-phonon interaction in [SbCl_5_]^2-^ octahedron^37,38^. The PL mechanism of CP2 crystals is shown in Fig. 2f.

### Scintillation performance

Under X-ray radiation, the high radiation energy firstly interact the heavy atoms of CP2 via Compton scattering and photoelectric effect, generating massive hot electrons. Subsequently, these electrons thermalise on an ultrafast timescale and combined with holes to form excitons. Then, the excitons interact with inorganic lattice and generate transient distortion due to the strong electron-phonon coupling, forming the excited state (STEs). At last, the excitons transient from STEs to the ground state.

**Fig. 3:**
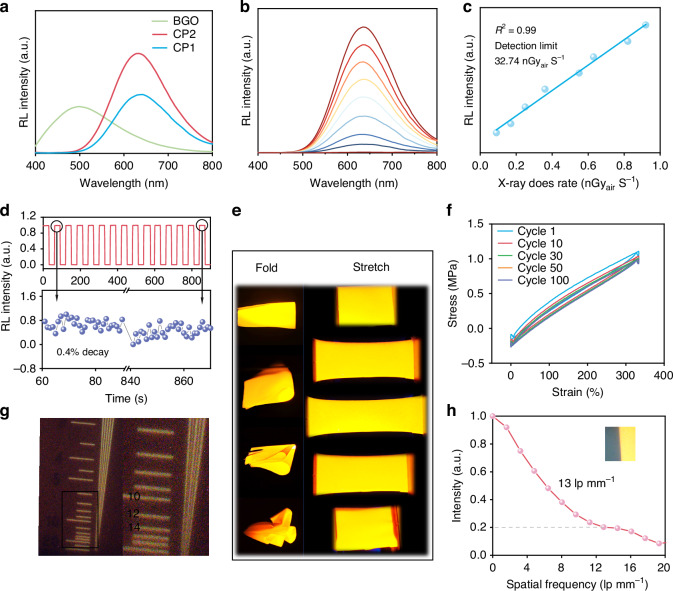
RL and flexibility characterizations of Sb^3+^-based OIHHs. **a** RL spectra of CP1 and CP2 crystals and reference scintillators; **b** X-ray excited luminescence (XEL) spectra of CP2 crystals under different X-ray dose rate (90.66–920.1 nGy_air_ s^–1^); **c** Detection limit of CP2 crystals; **d** X-ray irradiation resistance of CP2 crystals; **e** Flexible scintillator screen based on CP2 crystals under UV lights at different states; **f** Cyclic stretch of the flexible film; **g** X-ray images of a standard resolution test pattern plate based on CP2 powder film; **h** Modulation transfer function (MTF) curve of the scintillator screen

The scintillation performance of CP1 and CP2 crystals was characterized using X-ray relative light yields, X-ray dose dependences and irradiation resistance, respectively. The X-ray relative light yields plays an important role in real applications and is normally calculated with the commercial scintillators as references^39–42^. Figure 3a shows the radioluminescence spectra (RL) of CP1 and CP2 crystals and reference scintillators (BGO), and the X-ray light yields of CP1 and CP2 were calculated to be 19503 and 32332 photons MeV^–1^, respectively, which are 1.9 and 3.2 times higher than that of BGO (10,000 photons MeV^–1^). From Fig. S9, we can see that the values of X-ray absorption and X-ray attenuation efficiencies of CP1 and CP2 crystals are nearly the same due to their similar atomic composition. The X-ray dose dependence is considered as a crucial parameter in the field of medical imaging and the relationship between X-ray dose and RL intensity is linear within a certain dose range^38^. Ideal scintillators working well at low doses can reduce radiation exposure to patients and the standard medical diagnostics for X-ray imaging is 5.5 µGy_air_ s^–1^. Figure 3b shows that the RL intensity of CP2 crystals linearly increases with the X-ray dose from 90.6 to 920.1 nGy_air_ s^–1^ and the detection limit was 32.74 nGy_air_ s^–1^ (Fig. 3c) based on the signal-to-noise ratio at 3. Scintillators with the capability of high irradiation resistance can maintain stable light output under X-ray irradiation, which is crucial for detectors that need to work for a long time. Figure 3d displays the X-ray irradiation resistance of CP2 crystals, and shows a 0.4% decay after long-time X-ray irradiation of 780 s. The total X-ray dose rate was accumulated to 4.5 mGy_air_ s^-1^, indicating the reliability and effectiveness of scintillators. Besides that, the PL, RL and structural stabilities of CP2 crystals after six months storage in ambient conditions were investigated. Figures S10, S11 show that its PL and RL intensities and PXRD diffractions remain almost constant, showing the good environmental stability.

Based on the excellent photophysical and scintillation properties of CP2 crystals, they were ground into powders to fabricate a film using polymers (SEBS-g-MAH, SGM). The detailed preparation process is presented in “Methods”. Figure 3e shows the mechanical properties of the CP2 scintillator screen. The film can be folded, stretched and compressed multiple times with negligible damage. To further verify the flexibility of CP2 film, cyclic tensile tests were conducted. The Young’s modulus value of CP2 film was 1.14 MPa (Fig. S12), showing good deformation resistance. Cyclic tensile curves in Fig. 3f indicate that the film shows good shape memory after the repeated elongation for 100 cycles. The above results indicate that CP2 film exhibits excellent flexibility, which is suitable for non-planar X-ray imaging. Besides that, some other physical properties of the film were provided in Supporting Information. As shown in Fig. S13, the film with a size of 5 cm × 5 cm and a thickness of 200 μm exhibits high transparency.

In order to explore the X-ray imaging performance of CP2 film, a self-made planar X-ray imaging system (Fig. S14) was constructed. The spatial resolution of the flexible CP2 film was obtained with the help of an X-ray standard test card. As depicted in Fig. 3g, the spatial resolution is up to 14 lp mm^–1^, and the value is higher than most Sb^3+^-based OIHH films. As shown in Table S7, some main performance metrics of CP2 film even beyond the state-of-the-art scintillators. Besides that, the slanted edge method was used to verify the spatial resolution. When MTF is determined to be 0.2 (Fig. 3h), the final spatial resolution (13 lp mm^-1^) is close to the above result.

**Fig. 4:**
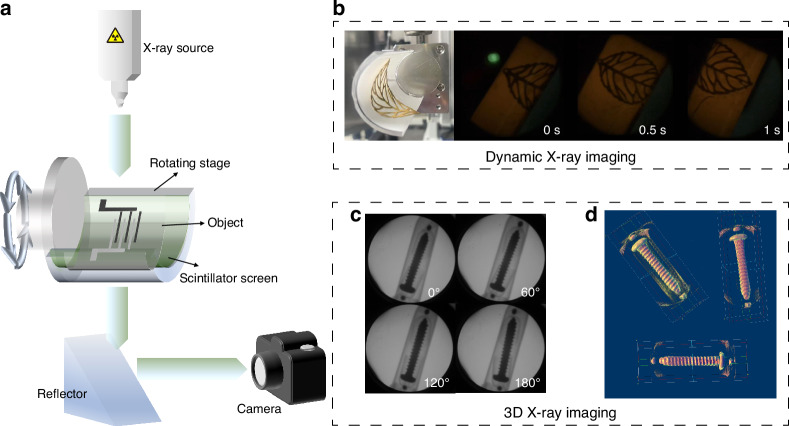
Dynamic and 3D X-ray imaging of CP2 film. **a** Schematic diagram of the rotatable X-ray imaging system; **b** Dynamic X-ray images of the curved copper ornament at different periods; **c** X-ray projection photos of a nail at partial rotation angles; **d** 3D reconstruction of a nail

Imaging objects (a conch and a peanut) with different X-ray absorption coefficients were placed on scintillator film and their internal structures can be clearly observed using our self-made planar X-ray imaging system under X-ray irradiation (Fig. S15), and the X-ray dose rate for X-ray imaging is 133 μGy_air_·s^–1^. To explore real-time dynamic X-ray imaging, a self-built 360° rotatable platform was created to rotate the objects (Fig. 4a). X-ray imaging items are a curved copper ornament and a flexible circuit, respectively, and the flexible CP2 film closely adheres to them to reduce image distortion resulting from the uneven spatial distribution of the X-ray dose. The X-ray projection photos of the circuit and copper ornament at different periods with the help of Nikon D850 were recorded, as shown in Fig. 4b and Fig. S16. The resolution was configured at 1080 p with a recording rate of 60 frames per second. In addition, as shown in Videos S1 and S2, the fine structures of the flexible circuit and copper ornament are clearly visible and there is no ghost or fake phenomenon, suggesting that the flexible film based on CP2 crystals successfully realized real-time dynamic imaging. Generally, some angles of 2D X-ray imaging cannot be detected, resulting in certain limitations in actual X-ray detection. Therefore, it is necessary to develop 3D X-ray imaging to solve these problems. We tried to collect the X-ray projection photos of a nail at different angles based on flexible CP2 film, and the angular scanning range was set from 0 to 180° with a step of 5° (Fig. S17). Some X-ray projection images of a nail at partial rotation angles are presented in Fig. 4c. Using the standard backpropagation algorithm (Feldkamp-Davis-Kress, FDK) for image reconstruction, a detailed 3D reconstruction of a nail was obtained (Fig. 4d), which greatly demonstrates the potential of Sb^3+^-based OIHHs for advanced X-ray imaging and detection. The corresponding code is open-sourced on GitHub to promote further research.

## Discussion

In this work, we designed and synthesized two Sb^3+^-based OIHHs (CP1 and CP2), which exhibit similar crystal structures except the introduction of water molecules in CP2 crystals. Both of them exhibit broadband orange emissions, large Stokes shifts and microsecond-level lifetimes, but CP2 crystals achieve a higher PLQY (97.25%) than that of CP1 (73.38%). We found that CP2 crystals have larger lattice distortion and smaller free degrees than that of CP1 due to the introduction of water molecules in CP2, which is easier for the formation of self-trapped excitons emissions and beneficial for light output. Benefitting from the high PLQY, CP2 crystals used as scintillators exhibit an impressive light yield of 32332 photons MeV^–1^, a low detection limit of 32.74 nGy_air_ s^–1^ and good X-ray irradiation resistance. Besides that, CP2 crystals were prepared into a flexible film, which demonstrated an exceptional spatial resolution of 14 lp mm^–1^. Furthermore, real-time dynamic and 3D X-ray imaging are realized with the help of the scintillator screen, which is the first time for Sb^3+^-based OIHH scintillators to realize these advanced X-ray imaging techniques. This work not only obtains novel Sb^3+^-based OIHH scintillators, but also broadens their applications for advanced X-ray detection and imaging.

## Materials and methods

### Synthesis of CP1 Crystals

CP1 crystals were prepared according to the following procedures. First, 1,3-bis(4-piperidyl) propane (0.1 mmol, 0.021 g) and SbCl_3_ (0.1 mmol, 0.023 g) were added into the mixed solution of HCl (2 mL) and ethanol (3 mL). The mixture was then heated to 110 °C for 2 h until complete dissolution. CP1 crystals were achieved by slowly cooling the reaction mixture to 60 °C at a rate of 10 °C/h.

### Synthesis of CP2 crystals

The synthesis procedure of CP2 is similar to that of CP1, except HCl was used as reaction solvents.

### Fabrication of CP2 film

Firstly, CP2 crystals (0.2 g) were ground into powders and were uniformly dispersed in 5 mL 0.1 g/mL toluene/(SEBS-g-MAH, SGM) solvent. Then, the mixed solution was continuously stirred for 12 h. Lastly, the homogeneous liquid was poured on glass sheet and slowly evaporated for 24 h at room temperature, resulting in the formation of a CP2 film.

### Structural characterization

The crystal structures for CP1 and CP2 crystals were determined on a Bruker Smart Apex CCD diffractometer with monochrome Mo-K radiation (λ = 0.71073 Å) source. The related PXRD tests were performed on an X-ray diffractometer D8 Advance A25. TG measurements were conducted on the NETZSCH STA-2500 synchronous thermal analyzer, with a heating rate of 10 °C/min in an air atmosphere. XPS tests were collected on a Shimazu Axis Supra spectrometer.

### Photoluminescence measurements

The photophysical properties of CP1 and CP2 crystals, including PLE spectra, PL spectra, emission lifetimes, and PLQYs, were measured using an Edinburgh Instruments FLS980 Spectrophotometer.

### Scintillation characterization

The scintillation performance, including RL spectra and linear X-ray response, of CP1 and CP2 crystals were obtained using a spectrofluorometer equipped with an X-ray source of an Au target. The light yields of CP1 and CP2 crystals were calculated with BGO as a reference. BGO powders were pressed into a mould with a diameter of 0.7 cm and a thickness of 0.1 mm. Based on the material’s density and a fixed volume, the mass of Sb^3+^-based halides was accurately determined. The samples were set at the same position to measure the XEL spectra, and the corresponding RL spectra were recorded by a spectrofluorometer. Finally, by integrating the steady-state XEL spectra, the light yields of CP1 and CP2 crystals were determined to be 19,503 and 32,332 photons MeV^–1^ respectively.

### Simulation section

The density functional theory (DFT) calculations for CP1 and CP2 crystals are achieved using the Vienna Ab-initio Simulation Package (VASP), employing the projector augmented wave (PAW) method. The relaxations for CP1 and CP2 crystals are performed with Gamma-only k-point, and a k-point mesh 2 × 2 × 2 with a separation of about 0.04 eV/Å is conducted for them. The energy cutoff for plane wave is set to 400 eV, the convergence energy criterion is set to 10^-5 ^eV and the convergence threshold is set to 0.02 eV/Å.

## Supplementary information

Supplementary Information for Highly Luminescent Organic-Inorganic Hybrid Antimony Halide Scintillators for Real-time Dynamic and 3D X-ray Imaging

Video S2

Video S1

## Data Availability

The code for 3D reconstruction is publicly available on GitHub at https://github.com/Grapeknight/matlab-fdk-reconstruction. The data supporting this study can be obtained from the corresponding author Q.Z. upon request.
