# Supplementary material for: Highly luminescent organic-inorganic hybrid antimony halide scintillators for real-time dynamic and 3D X-ray imaging
Source: Light Sci Appl. 2026 Jan 26;15:88. doi: 10.1038/s41377-025-02152-x (PMC12835138; doi:10.1038/s41377-025-02152-x)
Supplement: Supplementary file 1 — Supplementary Information for Highly Luminescent Organic-Inorganic Hybrid Antimony Halide Scintillators for Real-time Dynamic and 3D X-ray Imaging [file 41377_2025_2152_MOESM1_ESM.docx]

Supplementary Information for

Highly Luminescent Organic-Inorganic Hybrid Antimony Halide Scintillators for Real-time Dynamic and 3D X-ray Imaging

Haixia Cui,^1^ Wanjiao Li,^1^ Qianxi Li,^1^ Shaolong Wang,^1^ Mingye Zhu,^1^ Yongjing Deng,^1^ Shujuan Liu,^1^* and Qiang Zhao^1,2^*

^1^State Key Laboratory of Flexible Electronics (LoFE) & Institute of Advanced Materials (IAM), Nanjing University of Posts & Telecommunications, 9 Wenyuan Road, Nanjing 210023, Jiangsu, China.

^2^College of Electronic and Optical Engineering & College of Flexible Electronics (Future Technology), Jiangsu Province Engineering Research Center for Fabrication and Application of Special Optical Fiber Materials and Devices, Nanjing University of Posts and Telecommunications, 9 Wenyuan Road, Nanjing 210023, Jiangsu, China.

*Correspondence: iamsjliu@ njupt.edu.cn; iamqzhao@njupt.edu.cn

**Table S1.** Detailed single crystal X-ray data of CP1 and CP2.

| Identification code | CP1 | CP2 |
| --- | --- | --- |
| Chemical Formula | C_6.5_H_14_Cl_2.5_NSb_0.5_ | C_26_H_58_Cl_10_N_4_OSb_2_ |
| Formula Weight | 255.702 | 1040.76 |
| Crystal System | orthorhombic | orthorhombic |
| Space Group | Fddd | Fdd2 |
| *a* (Å) | 18.098(6) | 18.352(15) |
| *b* (Å) | 18.517(5) | 24.838(19) |
| *c* (Å) | 24.827(7) | 17.948(14) |
| *α* (^o^) | 90 | 90 |
| *β* (^o^) | 90 | 90 |
| *γ* (^o^) | 90 | 90 |
| *V* (Å^3^) | 8320(4) | 8181 |
| *Z* value | 32 | 8 |
| *D*(calcd) (g cm^-3^) | 1.633 | 1.690 |
| Temperature (K) | 293.15 | 293.19 |
| *F*(000) | 4099.5 | 4176.0 |
| *μ* (mm^-1^) | 1.965 | 2.001 |
| R_1_/wR_2_, [I>2σ(I)] | R_1_ = 0.0248, wR_2_ = 0.0574 | R_1_ = 0.0205, wR_2_ = 0.0500 |
| R_1_/wR_2_, [all data] | R_1_ = 0.0280, wR_2_ = 0.0602 | R_1_ = 0.0239, wR_2_ = 0.0525 |
| GOF | 1.080 | 1.206 |

**Table S2.** Selected bond lengths of CP1.

| Bond | Length/Å |
| --- | --- |
| Sb1-Cl3 | 2.5902 |
| Sb1-Cl3^1^ | 2.5902 |
| Sb1-Cl2^1^ | 2.6344 |
| Sb1-Cl2 | 2.6344 |
| Sb1-Cl1 | 2.3930 |

**Table S3.** Selected bond lengths of CP2.

| Bond | Length/Å |
| --- | --- |
| Sb1-Cl5 | 2.575 |
| Sb1-Cl2 | 2.575 |
| Sb1-Cl3 | 2.627 |
| Sb1-Cl1 | 2.617 |
| Sb1-Cl4 | 2.376 |

**Table S4.** Selected bond angles of CP1.

| Bond | Angle/° |
| --- | --- |
| Cl3^1^ Sb1 Cl3 | 179.37(3) |
| Cl2 Sb1 Cl3 | 87.12(2) |
| Cl2 Sb1 Cl3^1^ | 92.82(2) |
| Cl2^1^ Sb1 Cl3 | 92.82(2) |
| Cl2^1^ Sb1 Cl3^1^ | 87.12(2) |
| Cl2^1^ Sb1 Cl2 | 170.59(4) |
| Cl1 Sb1 Cl3 | 89.685(16) |
| Cl1 Sb1 Cl3^1^ | 89.685(16) |
| Cl1 Sb1 Cl2 | 85.294(18) |
| Cl1 Sb1 Cl2^1^ | 85.294(18) |

**Table S5.** Selected bond angles of CP2.

| Bond | Angle/° |
| --- | --- |
| Cl5 Sb1 Cl3 | 87.02 |
| Cl5 Sb1 Cl1 | 93.04 |
| Cl2 Sb1 Cl1 | 86.96 |
| Cl2 Sb1 Cl3 | 92.74 |
| Cl4 Sb1 Cl3 | 85.20 |
| Cl4 Sb1 Cl5 | 89.31 |
| Cl4 Sb1 Cl2 | 89.33 |
| Cl4 Sb1 Cl1 | 84.80 |
| Cl5 Sb1 Cl2 | 176.83 |
| Cl1 Sb1 Cl3 | 170.01 |

**Table S6.** Summary of the photophysical parameters for CP1 and CP2.

|  | CP1 | CP2 |
| --- | --- | --- |
| *Ex* (nm) | 365 | 365 |
| *Em* (nm) | 632 | 642 |
| *τ* **(**μs**)** | 5.19 | 5.16 |
| PLQY (%) | 73.38 | 97.25 |
| FWHM (nm) | 144 | 146 |
| Stokes (nm) | 267 | 277 |
| *k_r_* (μs^-1^) | 0.1414 | 0.1885 |
| *k_nr_* (μs^-1^) | 0.0513 | 0.0053 |

**Table S7.** Summary of the scintillation performance for the state-of-the-art scintillators.

| Compound | Lifetime  (μs) | PLOY  (%) | Light yields  (photons MeV^-1^) | Resolution  (lp mm^-1^) | Ref. |
| --- | --- | --- | --- | --- | --- |
| CsPbBr_3_ QDs | 0.045 | 58.6 | 30240 | 7 | [1] |
| MAPbBr_3_ QDs | 0.04 | >31.2 | - | 9 | [2] |
| BA_2_PbBr_4_: Mn | 727.83 | 57.58 | 85000 | 10.7 | [3] |
| MCy-Mn-Glass | 355.3 | >90 | 58662 | 19.3 | [4] |
| (he-ted)_2_MnBr_4_-Glass | 206.24 | 99 | 20784 | 20.4 | [5] |
| Cs_3_Cu_2_I_5_-AAO | 0.936 | 59.9 | 29600 | 10.4 | [6] |
| Cs_2_Ag_0.6_Na_0.4_In_0.85_Bi_0.15_Cl_6_ | 2.8 | 90 | 39000 | 4.3 | [7] |
| Cu_4_I_6_(pr-ted)_2_ | 8.0 | 97.1 | 32600 | 20 | [8] |
| BGO | 0.3 | <10 | 10000 | - | [9] |
| TEBA-2 | 2.04 | 73.83 | 15000 | 10.4 | [10] |
| (PPN)_2_SbCl_5_ | 4.1 | 98.1 | 49000 | - | [11] |
| [Na(DMSO)_2_]_3_SbBr_3_Cl_3_ | 6.5 | 90.6 | - | 15.5 | [12] |
| [Bmmim]_2_SbCl_5_ | 8.9 | 80.82 | 9987 | 12.5 | [13] |
| [FPPP]_2_SbCl_7_ | 6.49 | 99.26 | 46927 | 13.01 | [14] |
| (NYP)_2_SbCl_5_ | 0.33 | 75.07 | 21500 | 7.77 | [15] |
| (C_20_H_20_P)_2_SbCl_5_-Glass | 3.58 | 44.5 | 12535 | 30 | [16] |
| [Ga(DMSO)_6_][SbCl_6_] | 1.17 | 93.7 | 12904 | 6 | [17] |
| (ETP)_2_SbCl_5_ | 1.074 | 99.3 | 8500 | 19 | [18] |
| (MTP_2_SbCl_5_) | 3.61 | 99.69 | 39800 | 10.2 | [19] |
| C_38_H_36_P_2_Sb_2_Cl_8_ | 3.59 | 99.8 | 41300 | 8.15 | [20] |
| C_50_H_44_P_2_SbCl_5_ | 2.67 | 98.42 | 44460 | 8.2 | [21] |
| (BPP)_2_SbCl_5_ 0.5 H_2_O | 5.16 | 97.25 | 32332 | 13-14 | This work |

**Fig. S1** (a) X-ray photoelectron spectroscopy (XPS) of CP1. High-resolution spectra of (b) Sb 3d; (c) Cl 2p and (d) N 3d.

**Fig. S2** High-resolution spectra of (a) Sb 3d; (b) N 3d; (c) Cl 2p and (d) O 1s.

**Fig. S3** Thermogravimetric analysis curves of CP1 and CP2 crystals.

**Fig. S4** (a) Electronic band structure and (b) density of states of CP1 crystals; (c) VBM (left) and CBM (right) of CP1 crystals.

**Fig. S5** (a) Absorption spectra of antimony-based OIHHs; The energy gaps of (b) CP1 and (c) CP2.

**Fig. S6** PLQYs of antimony-based OIHHs (a) CP1 and (b) CP2.

**Fig. S7** The functional plot between PL intensity and temperature.

**Fig. S8** The functional plot between FWHM and temperature.

**Fig. S9** (a) The absorption coefficients versus X-ray photon energy; (b) The curves of the attenuation efficiency versus thickness; (c) The curves of the attenuation efficiency versus X-ray photon energy for BGO, CP1 and CP2.

**Fig. S10** Photographs of CP2 crystals for different times under daylight, UV-365 nm and X-ray irradiation, respectively.

**Fig. S11** The PXRD patterns of CP2 crystals exposed to air at different periods.

**Fig. S12** (b) Tensile stress-strain curves of the flexible film.

**Fig. S13** Schematic diagram of the planar X-ray imaging system.

**Fig. S14** Schematic diagram of the planar X-ray imaging system.

**Fig. S15** Bright-field (left) and X-ray images (right) of a conch and a peanut.

**Fig. S16** Dynamic X-ray images of a flexible circuit.

**Fig. S17** X-ray projection photos of a nail at different angles from 0 to 180° with a step of 5°.

**References**

1. Chen, Q. S. et al. All-inorganic perovskite nanocrystal scintillators. *Nature* **561**, 88-93 (2018).
2. Xu, Q. et al. Ultra-flexible and highly sensitive scintillation screen based on perovskite quantum dots for non-flat objects X-ray imaging. *Materials Today Physics* **18**,100390 (2021).
3. Shao, W. Y. et al. Highly efficient and flexible scintillation screen based on manganese (II) activated 2D perovskite for planar and nonplanar high-resolution x-ray imaging. *Advanced Optical Materials* **10**, 2102282 (2022).
4. Zhou, Z. J. et al. Cationic Substituent Engineering to Enhance Glassy Stability of Manganese Halide Scintillators for Advanced 3D X-ray Reconstruction. *Laser* *&* *Photonics* *Reviews* **19**, 2500657 (2025).
5. Zhou, Z. J. et al. Custom-Shapable and Reusable Thermoplastic Manganese Halide Scintillation Glass for Curved X-Ray Imaging. *Laser* *&* *Photonics* *Reviews* **19**, 2401489 (2025).
6. Zhao, X. et al. Embedding Cs3Cu2I5 scintillators into anodic aluminum oxide matrix for high-resolution X-ray imaging. *Advanced Optical Materials* **9**, 2101194 (2021).
7. Zhu, W. J. et al. Low-dose real-time X-ray imaging with nontoxic double perovskite scintillators. *Light: Science & Applications* **9**, 112 (2020).
8. Wang, Y. Z. et al. Efficient X-ray luminescence imaging with ultrastable and eco-friendly copper (I)-iodide cluster microcubes. *Light: Science & Applications* **12**, 155 (2023).
9. Cui, H. X. et al. Rod-Like Microcrystal Scintillators Based on Organic-Inorganic Hybrid Copper Halides for X-Ray Imaging. *Laser* *&* *Photonics* *Reviews* **19**, 2401321 (2025).
10. Chen, H. Y. et al. Highly luminescent antimony-based organic–inorganic hybrid halides for X-ray imaging and detection. *Journal* *of* *Materials* *Chemistry* *C* **12**, 12325-12331 (2024).
11. He, Q. Q. et al. Highly stable organic antimony halide crystals for X-ray scintillation. *ACS Materials Lett*ers **2**, 633-638 (2020).
12. Mo, Q. H. et al. High quantum efficiency of stable Sb-based perovskite-like halides toward white light emission and flexible X-ray imaging. *Advanced Optical Materials* **10**, 2201509 (2022).
13. Li, H. et al. A thermoplastic organic metal halide scintillator. *ACS Materials Letters* **5**, 2481-2487 (2023).
14. Wang, Y. Y. et al. A lead-free zero-dimensional hybrid antimony halide perovskite X-ray scintillator with exceptional emission efficiency and excellent stability as a highly sensitive fluorescent probe. *Inorganic Chemistry Frontiers* **11**, 5034-5042 (2024).
15. Cao, S. J. et al. Tunable luminescence based on structural regulation in organic antimony halides for X-ray scintillation. *Inorganic Chemistry Frontiers* **11**, 5221-5232 (2024).
16. Feng, T. et al. Large-area transparent antimony-based Perovskite Glass for High-Resolution X-ray imaging. *ACS Nano* **18**, 16715-16725 (2024).
17. Shi, H. Y. et al. Regulating the Liquid-to-Solid Transition of a Solvent-Coordinated Metal Halide for Low-Temperature-Processed Pixelated Scintillators. *Inorganic Chemistry* **64**: 9386-9391 (2025).
18. Xu, Z. W. et al. Transparent 0D antimony halides glassy wafer with near-unity photoluminescence quantum yield for high spatial resolution X-Ray imaging. *Advanced Optical Materials* **12**, 2301477 (2024).
19. Meng, H. X. et al. Highly efficient flexible antimony halide scintillator films with in situ preparation for high-resolution X-Ray imaging. *Laser* *&* *Photonics* *Reviews* **19**, 2401703 (2025).
20. Meng, H. X. et al. Stable Organic-Inorganic Hybrid Sb (III) Halide Scintillator for Nonplanar Ultra-Flexible X-Ray Imaging. *Advanced Functional Materials* **35**, 2412597 (2025).
21. Meng, H. X. et al. Stable Organic Antimony Halides with Near-Unity Photoluminescence Quantum Yield for X-Ray Imaging. *Laser* *&* *Photonics* *Reviews* **17**, 2201007 (2023).
